# Effervescence-Assisted Microextraction—One Decade of Developments

**DOI:** 10.3390/molecules25246053

**Published:** 2020-12-21

**Authors:** Guillermo Lasarte-Aragonés, Rafael Lucena, Soledad Cárdenas

**Affiliations:** Departamento de Química Analítica, Instituto Universitario de Investigación en Química Fina y Nanoquímica (IUNAN), Universidad de Córdoba, Campus de Rabanales, Edificio Marie Curie (anexo), 14071 Córdoba, Spain; b22laarg@uco.es (G.L.-A.); rafael.lucena@uco.es (R.L.)

**Keywords:** dispersion, micro-solid phase extraction, dispersive liquid–liquid extraction, effervescence

## Abstract

Dispersive microextraction techniques are key in the analytical sample treatment context as they combine a favored thermodynamics and kinetics isolation of the target analytes from the sample matrix. The dispersion of the extractant in the form of tiny particles or drops, depending on the technique, into the sample enlarges the contact surface area between phases, thus enhancing the mass transference. This dispersion can be achieved by applying external energy sources, the use of chemicals, or the combination of both strategies. Effervescence-assisted microextraction emerged in 2011 as a new alternative in this context. The technique uses in situ-generated carbon dioxide as the disperser, and it has been successfully applied in the solid-phase and liquid-phase microextraction fields. This minireview explains the main fundamentals of the technique, its potential and the main developments reported.

## 1. Introduction

The efficacy of a given microextraction technique is controlled by both thermodynamic and kinetic factors. The thermodynamics defines the total amount of analyte that can be isolated from the sample, while kinetics defines the rate at which the mass transference equilibrium is achieved [1]. These miniaturized techniques usually work under diffusion-controlled conditions due to the differences in size between the donor solution (sample) and the acceptor (extractant) phase, among other aspects. This difference makes the diffusion distances large, thus reducing the extraction rate. From a practical point of view, a thermodynamically favored microextraction is only useful when it is rapid enough as the sample throughput is essential to respond to the growing demand for analytical information [2]. Thus, kinetics factors are significant in the development of new microextraction techniques.

The contact surface area between the donor and the acceptor solution is, in the context of the present article, one of the critical kinetics variables [3]. The dispersion of the extractant phase into the sample in the form of tiny particles or drops is the most common approach to boost the contact surface area [4]. Several strategies, both in the solid-phase and liquid-phase microextraction contexts, have been proposed. These strategies can be generally divided into two main groups depending on the use of external energy [5,6] or chemicals [7,8] to achieve this dispersion.

In this article, effervescence-assisted microextraction is reviewed. This technique was initially proposed by our group in 2011 [9] and, since then, it has been developed and applied by many different groups worldwide. The technique is based on the in situ generation of carbon dioxide because of the reaction between a carbon dioxide donor and a proton donor. The gas generated produces an efficient dispersion of the extractant phase into the sample. The technique has been successfully applied to the solid-phase and liquid-phase microextraction contexts, and the main developments are described in the next sections.

## 2. Effervescence-Assisted Dispersive Micro-Solid Phase Extraction

Dispersive solid phase extraction (DSPE) is based on the dispersion of a solid sorbent into the sample of interest. Anastassiades introduced this approach as a fast and simple method for the determination of pesticides in complex foodstuff matrices [10]. This first proposal was used as clean-up method aimed to remove interferences from the sample matrix by adding a certain amount of sorbent assisted by vortex agitation. The sorbent is then dispersed to enhance the contact surface with the solution, cleaning it from interferents but maintaining target analytes in solution. This kind of cleanup strategy later received the name of QuEChERS, an acronym for its advantages: quick, easy, cheap, effective, rugged, and safe. Nowadays, it is commercially available as sample treatment strategy and it has been extensively applied to a variety of matrices and analytes [11].

The application of dispersive methodologies using reduced amount of sorbents provides high efficiency and enrichment factors in a shorter procedure time [12]. The miniaturized technique is usually named as dispersive micro-solid phase extraction (DµSPE). The cornerstone of DµSPE is the dispersion of the sorbent (extractant phase) in the sample. The process must take into consideration the nature and properties of the sorbent (polarity, micro or nano-size, aggregation) and can be achieved by physical or chemical means. Physical dispersion is typically assisted by an external energy source such as ultrasound irradiation [13] or vortex agitation [14]. Chemical dispersion is aimed to improve dispersibility of the sorbent by means of water-miscible organic solvent such as acetonitrile or methanol [15].

**Figure 1:**
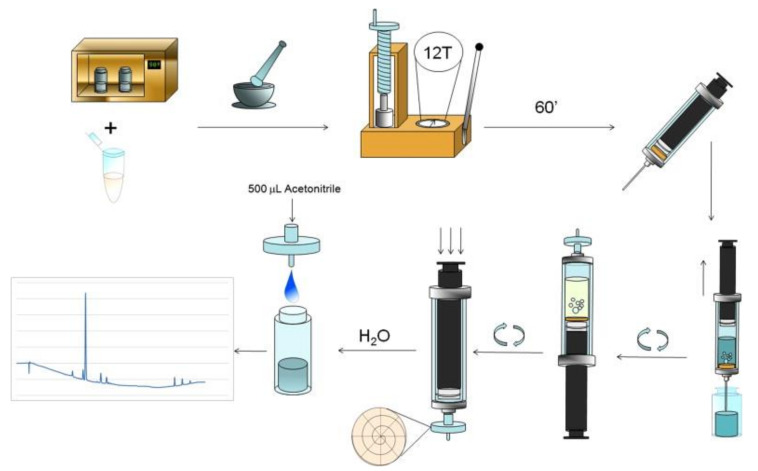
Description of the general analytical procedure of the effervescence-assisted dispersive micro-solid phase extraction. Reproduced with permission of Elsevier from reference [9].

In 2011, our research group introduced a new format of DµSPE based on in situ-generated CO_2_ by an effervescent reaction [9]. This new effervescent-assisted alternative is based on the fabrication of a tablet containing a commercial sorbent (OASIS-HLB) and reaction precursors (sodium carbonate as CO_2_ source and sodium dihydrogen phosphate as proton donor). The tablet containing all the elements was then introduced into the aqueous sample and the sorbent is effectively dispersed by means of the in situ-generated gas bubbles. The alternative is designed to provide all the elements to perform the extraction process on-site, avoiding the use of disperser solvent (minimizing environmental impact and waste generation) or external apparatus (such as vortex or ultrasounds). In fact, the first implementation of the technique employs a syringe as sample-collection and extraction vessel device. The effervescent sorbent tablet is placed inside the syringe and the extraction process start once the sample is aspirated. The dispersed sorbent can be easily recovered by means of a syringe filter and eluted before analysis. The complete process is depicted in Figure 1. The method was employed for the determination of nitroaromatic compounds in water samples. Its analytical performance was comparable to other SPE alternatives for the same analytical problem, but in a simpler and rapid fashion. Analyte partition equilibrium is not affected by the effervescence process and different sorbents can be used according to the target analytes.

### 2.1. Use of Effervescent Tablets for EA-DμSPE

The potential of effervescent tablets was later demonstrated in the effective dispersion of a nanometric sorbent, multiwalled carbon nanotubes (MWCNTs) in aqueous matrices [16]. Carbon nanostructures have been widely used in analytical nanoscience because of their properties as sorbents (large surface area, different interaction mechanism with target analytes); however, their dispersibility in aqueous phase is limited given their trend to aggregation unless they are modified or immobilized [17]. The use of effervescent tablet (102 mm ID) was responsible for the efficient dispersion of the unmodified sorbent and not the salts, resulting much more effective than mechanical agitation. By this means, a small amount of sorbent (7.5 mg) can be successfully dispersed in a large sample volume (100 mL) without the assistance of any external apparatus or energy source. The proposed alternative combined with Liquid Chromatography-Diode Array Detector (LC-DAD) was comparable with other alternatives in terms of sensitivity and precision but reducing the processing time to minutes. Later, Wang and co-workers fabricated effervescent tablets (102 mm ID) with MWCNTs as sorbent for the extraction of natural antioxidants in hawthorn samples (medicinal and food plants) before LC-ECD (Electron Capture Detector ) analysis [18]. Authors selected 300 mg of effervescent precursors (NaH_2_PO_4_ and Na_2_CO_3_) as the optimum value, since higher amounts could have a negative impact on the extraction efficiency by increasing the ionic strength and viscosity of the sample and subsequently reducing the mass transfer process. Different carbon nanostructured sorbents were evaluated in terms of their extraction performance, including unmodified, hydroxylated and carboxylated CNTs. Authors selected carboxy-graphited MWCNTs over pristine MWCNT or hydroxyl-graphited MWCNTs because they present stronger interaction with the target analytes, and, therefore, its extraction capacity was superior.

Ye et al. proposed the use of mesoporous hybrid materials as sorbent in combination with effervescent tablet for the determination of tanshinone compounds from herbal preparations by LC-DAD [19]. The sorbent, permanent confined micelle arrays-60 (PCMA-60), has a highly ordered structure with mesoporous channels with great extraction potential for hydrophobic analytes. The dispersion capacity of effervescent tablets (102 mm ID) seems to overcome the aggregation trend of PCMA-60 due to hydrophobic interaction. However, to achieve a good analytical performance and tablet mechanical endurance, sorbent amount must be below 13 mg.

**Figure 2:**
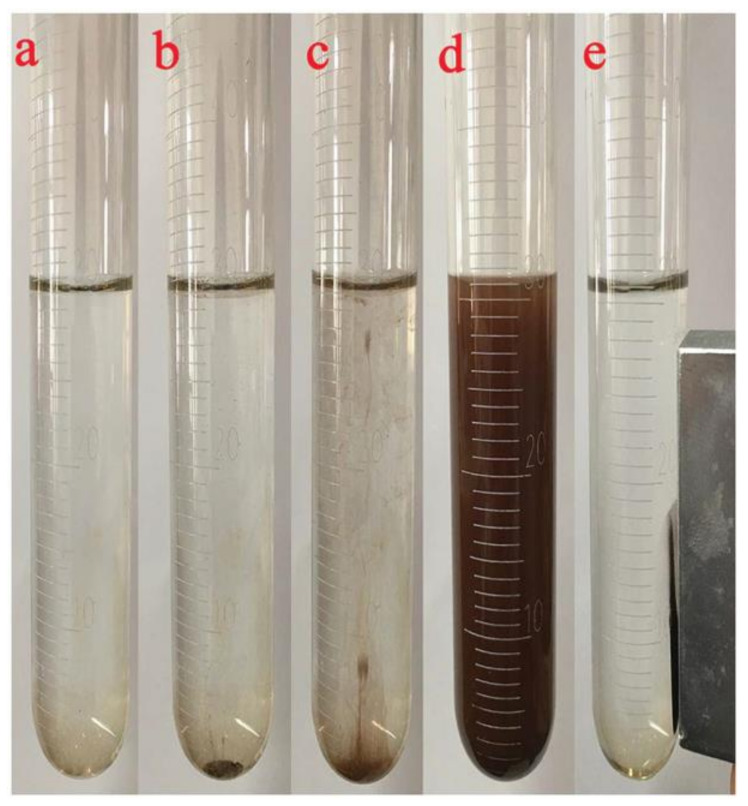
(**a**) A total of 30 mL of the pretreated sample is added into a 50 mL glass tube; (**b**) magnetic effervescent tablet is added into the tube and the effervescent reaction takes place; (**c**) effervescence could be maintained for 3 min until (**d**) complete dispersion of the sorbent is observed; (**e**) finally, by means of an external magnet the sorbent, enriched with heavy metal ions, is converged and isolated. Reproduced with permission of Royal Society of Chemistry from reference [24].

Yang et al. designed an effervescent tablet containing a composite of BCD/ATP (β-cyclodextrin/attapulgite) for in-syringe extraction of pyrethroids from water samples [20]. The combination of the BCD inclusion properties and the structure of ATP (a 2D crystalline magnesium silicate) allows the efficient on-site enrichment of the target analytes without assistance of any external apparatus. The composite can be easily recovered from the syringe by means of a 0.22 μm filter coupled to the extraction vessel. Moreover, its analytical performance is comparable to other alternatives that require ultrasound assistance or centrifugation on a very simple procedure. Recently, they suggested a combination of attapulguite and polypyrrole with magnetic nanoparticles (MNPs) on an effervescent tablet for the same target analytes in honey samples [21]. The same research group took one step forward developing a magnetic BCD/ATP composite with surface modification with ionic liquids (IL) [22]. This new sorbent was combined with effervescent precursors (Na_2_CO_3_ and NaH_2_PO_4_) and employed in the extraction of fungicides from food samples. The sorbent recovery is facilitated by its magnetic properties, whereas the IL modification enhances the overall extraction capacity [23]. Precisely, magnetic sorbent-recovery has aroused impact as an alternative to filtering or centrifugation in microextraction procedures. Zhou and co-workers prepared an effervescent tablet containing NiFe_2_O_4_ nanoparticles (NPs) for the preconcentration of heavy metal traces from seafood samples [24]. The tablet (8 mm ID × 2 mm thickness) is introduced in the extraction vessel containing 30 mL of pre-treated foodstuff aqueous solution. The dispersion of the nanostructured sorbent is completed during the effervescent reaction and the recovery is carried out by a magnet on the side of the extraction vessel (Figure 2). Electrostatic attraction of metal cations (Cu, Cd, Zn, and Mn) to the NPs surface combined with its large surface area avoid the utilization of chelating agents. The microextraction method combined with Inductively Coupled Plasma-Mass Spectrometry (ICP-MS) allowed the determination of the target metals at the low μgkg^−1^ level, which is more than 300-fold lower than other alternatives. In the work of Fahimirad, heavy metals (Pb, Cd, Ni and Cu) are determined by means of an effervescent tablet containing a magnetic nanosorbent [25]. The designed composite consisted of chitosan-Fe_3_O_4_ nanoparticles with immobilized diphenyl diselenide groups to enhance complex formation capacity. The sorbent (8 mg) was later compressed with effervescent precursors (Na_2_CO_3_ and citric acid) in tablet format (10 mm ID × 5 mm thickness) for the EA-DSPE of heavy metal traces by flame atomic absorption spectrometry from food and water samples. The proposed alternative is particularly fast (only 30 s of extraction time) and can be reused up to four times. Karbalaie recently suggested the use of effervescent tablets (10 mm ID × 5 mm thickness) containing dopamine-modified sorbents for the EA-DµSPE of metals from water and foodstuff samples [26,27]. In particular, the modification of magnetic graphene-oxide with dopamine in this format showed a high preconcentration factor (333-fold) with extended reusability (up to 5 times for a 10 mg sorbent dosage) thanks to the enhanced extraction capacity provided by dopamine modification [28].

A newly proposed magnetic sorbent, with nitrogen-rich surface modification (Fe_3_O_4_@SiO_2_@N_3_), was also combined with effervescent dispersion by the same research group [29]. The sorbent was successfully applied for the extraction of antidepressants from urine and pharmaceutical wastewater samples. The surface modification enhanced the overall extraction capacity on a very fast format (1 min extraction time) thanks to the efficient effervescent dispersion in the aqueous phase. Moreover, when compared with previously published method, the effervescent-assisted alternative combined with LC-UV (Ultraviolet detector) analysis resulted on the fastest, precise, and sensible method. Another example of extraction efficiency enhancement by surface modification is presented in the work by Ding and co-workers [30]. In this work, a tablet containing magnetic nickel-based nitrogen doped graphene tubes (Ni@N-GrTs) was compressed with the effervescence precursors (Na_2_CO_3_ and tartaric acid) to form a tablet (8 mm ID × 1 mm thickness). The tablet was then introduced in deproteinized and water-diluted milk samples for the extraction and determination of trace bisphenols by LC-FLD (Fluorescence detector). Interestingly, the developed sorbent presents a series of structural defects and reactive sites aimed to enhance the overall extraction capacity (by increasing π–π interaction and H-bond with the target analytes). The dispersion of the sorbent by effervescent reaction was complete in less than 3 min, and once recovered, the sorbent can be washed and reused on a new extraction cycle. The same analytes and other endocrine disruptors were determined by Tan et al. in water samples using effervescent tablets containing core-shell magnetic organic frameworks (NiFe_2_O_4_@COF) as sorbents [31].

An interesting modification of the effervescent-assisted procedure was presented by Hu [32]. The method consisted of an effervescence-assisted matrix solid-phase extraction using crown ether (benzo-15-crown-5) as sorbent. To this aim, dried *C. fraxini* (medicinal herb) was blended with effervescent precursors (NaH_2_PO_4_ and NaHCO_3_) and sorbent and compressed into tablet form. The tablet was introduced into a vial containing 2.5 mL of water; once the effervescence finished, the herb debris were centrifuged, and the solution was filtered before UHPLC analysis. The target analytes are four coumarins (esculin, esculetin, fraxin and fraxetin) and were extracted using the cavity and hydrophobic properties of the crown ether.

### 2.2. Other Formats for EA-DμSPE

**Figure 3:**
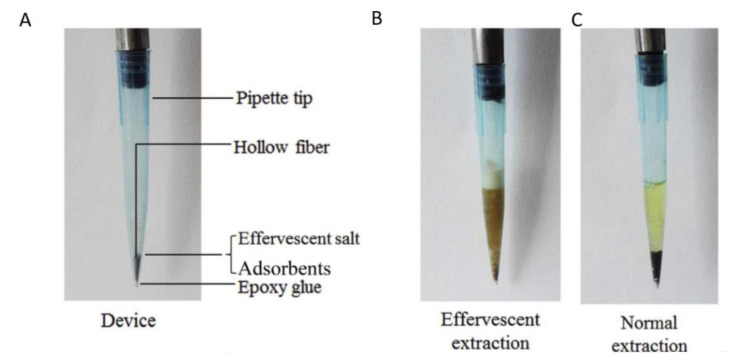
(**A**) Effervescence-Pippete Tip SPE device (EPT-SPE); (**B**) Effervescent extraction; (**C**) Normal extraction in absence of effervescent precursors. Reproduced with permission of Elsevier from reference [33].

Although tableting is the most extended format to achieve effervescent-assisted dispersion, other alternatives generate CO_2_ bubbles are available. Wang and coworkers designed a pipette tip containing a carbon nanotube-polystyrene-divinylbenzene composite and NaHCO_3_ (Figure 3A) [33]. The sample is mixed with the second effervescence precursor, NaH_2_PO_4_, and once it is pipetted the effervescence reaction takes places inside the tip and the sorbent is efficiently dispersed (Figure 3B). The device was employed in the determination of natural alkaloids in biological samples (urine, feces, and cell culture). The sorbent is efficiently dispersed only if effervescence takes place inside the pipette tip (Figure 3C) This configuration offer advantages over the tablet format, in particular the reduction of the sample volume and effervescent precursors (500 μL of sample can be extracted by 10 mg of sorbent dispersed by means of 3 mg of NaHCO_3_) and the simplicity of storage conditions and powder preparation.

Jamshidi and co-workers used an IL (1-butyl-3-methylimidazolium hexafluorophosphate, [BMIM] [PF_6_])-coated magnetic core-shell nano particles for effervescence powder-assisted µSPE for the extraction of betablockers from plasma samples [34]. The authors compared two different extraction procedures, in one hand the magnetic nanoparticles and [BMIM] [PF_6_] are homogeneously blended with effervescent precursors (namely magnetic effervescent powder or MEP) and used for the procedure; on the other, the IL is immobilized on the MNPs surface and added simultaneously with 50 mg of effervescence precursors (NaH_2_PO_4_ and NaHCO_3_) to the sample solution to perform the extraction. The results showed that the immobilized IL dispersed by means of effervescent powder the most successful alternative. The effervescence generated by means of the so-called MEP separates the IL and makes harder to recover from solution. Moreover, when compared with other microextraction alternatives this method combined with LC-MS (Mass spectrometry) analysis is one of the most sensitive.

## 3. Effervescence-Assisted Dispersive Liquid-Phase Microextraction

Dispersive liquid–liquid extraction (DLLME), proposed by Rezaee et al. in 2006, is based on the efficient dispersion of an extractant solvent into the sample [35]. In the typical approach, this dispersion is aided by a disperser solvent that is miscible with both the sample and the extractant phase. The extraction takes place in several and consecutive steps, namely: (a) the sample is placed in an extraction vessel, typically a centrifugation tube; (b) a mixture of the disperser and extractant solvents are rapidly injected into the sample using a syringe; (c) the solubilization of the disperser solvent into the sample release tiny drops of extractant that interact with the target analytes; (d) the extractant solvent is coalesced and recovered by centrifugation. DLLME provides higher extraction recoveries than most liquid phase microextraction techniques (LPME) due to the improvement of the mass transference. In fact, many LPME techniques work under the kinetic range, where the extraction recovery dramatically depends on the time, and in most cases, this efficiency must be sacrificed to improve the sample throughput. However, in DLMME, the partitioning equilibrium is achieved almost instantaneously, providing an optimum extraction.

The use of an organic solvent as the disperser has two main shortcomings. On the one hand, the volume of disperser solvent is in the mL range, and, therefore, DLLME cannot be completely considered a microextraction technique. On the other hand, the disperser solvent is mixed with the aqueous sample, increasing the analytes’ solubility in the donor phase. This aspect clearly affects the distribution constant reducing the transference of the analytes. Avoiding the disperser solvent or its negative effect on the analytes partition has been the focus of intensive research in the last decade.

**Figure 4:**
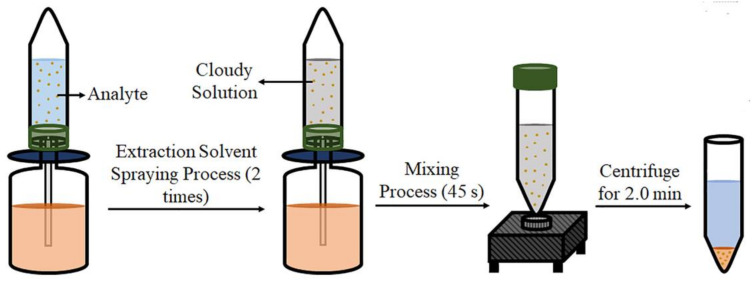
Schematic diagram of the vortex-assisted spraying-based fine droplet formation liquid-phase microextraction. Reproduced with permission of Wiley from reference [39].

The disperser solvent can be reduced or even avoided if an external energy source, like US [36] and vortex [5], is used. These sources have similar advantages and disadvantages to those described for d-µSPE. Additionally, this external energy can be provided in other ways. In the so-called air-assisted liquid–liquid extraction (AALLME) [37], the disperser solvent is wholly avoided. In AALLME the donor-acceptor mixture is repeatedly aspirated and dispensed with a syringe producing a somewhat mechanical mixing [38]. Recently, Bakirdere and coworkers have proposed an innovative approach based on a nasal sprayer (Figure 4). The sprayer container was filled with the extraction solvent, and a centrifuge tube is adapted to the container by a special cap [39]. When pushed, the sprayer releases the extraction solvent as a cloud of tiny drops. The tube was stirred for 45 s to increase the mass transference, and the extracts were finally recovered by centrifugation.

Gas can also be used as a disperser agent since it does not affect the partition of the analytes between phases and can be easily purged from the solution after the extraction. Raterink et al. proposed the gas pressure-assisted extraction where a gas stream is bubbled in the sample-extractant biphasic system for efficient mixing [40]. Other authors preferred the use of special devices to nebulize the extractant solvent into the sample. In this sense, Sun et al. reported the use of an inkjet nebulizer as a way to disperse a low volume of organic solvents (10 µL) in the form of tiny drops (20 µm in size) using air as the disperser [41].

**Figure 5:**
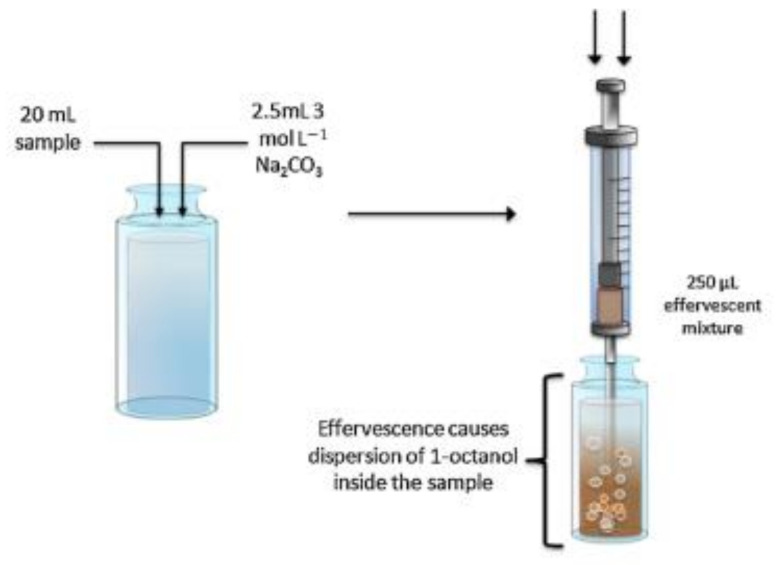
Schematic diagram of the effervescence-assisted dispersive liquid–liquid extraction (DLLME). Adapted with permission of Elsevier from reference [42].

Our group proposed the adaptation of effervescence extraction to the LPME context in 2014 [42]. Effervescence-assisted DLLME (EA-DLLME) consists of the in situ generation of carbon dioxide to promote the close contact between the donor and the acceptor phases. As it is indicated in Figure 5, sodium carbonate is added to the sample, and subsequently, the extractant solvent, mixed with acetic acid, is rapidly injected. The reaction between the acetic acid and sodium carbonate generates carbon dioxide (the disperser) and sodium acetate that contributes to the ionic strength and may produce a salting-out effect. In this preliminary work, magnetic nanoparticles were added to the acetic acid-extractant mixture and used to recover the extractant from the sample avoiding the centrifugation process.

Since the description of EA-DLLME, many valuable contributions have been reported extending its applicability by modifying the extraction workflow and using novel solvents.

### 3.1. Extraction Workflows

#### 3.1.1. Use of Effervescence Tablets for EA-DLLME

In contrast with the first EA-DLLME approach [42], most of the successive applications used a tablet for the administration of all or some of the effervescent agents. In 2014, Jiang et al. applied this approach for the first time to extract some fungicides from apple juice [43]. For this purpose, a tablet containing potassium carbonate and citric acid was placed in the extraction tube for the dispersion of the organic solvent (chlorobenzene) into the sample. After the extraction, the solvent was recovered by centrifugation. From this initial work, several strategies have been reported for the fabrication of the tablets. For better understanding, they can be classified according to number of key ingredients (carbon dioxide source, H-donor and extractant) into tertiary or binary mixtures. As it will be commented on, other additives are added to improve the tablet performance.

Yıldız and Çabuk proposed a tablet based on a tertiary mixture containing Na_2_CO_3,_ NaH_2_PO_4_ and 1-dodecanol [44] for the extraction of fungicides from fruit juice. For the fabrication, all the ingredients were manually blended and cooled in a refrigerator to produce the solidification of the 1-dodecanol increasing the consistency of the tablet. To achieve a reproducible shape and size, the tablets were fabricated using an empty pill strip as mold. The tablets can be immediately used or stored in the fridge until their final application. In the extraction workflow the appropriate temperature control is critical. During the extraction, the sample is heated at 50 °C to maintain the extractant solvent in its liquid form enhancing its dispersion. After the extraction, the solution is centrifuged and cooled down to recover 1-dodecanol as a solid. A very similar fabrication approach but using 1-undecanol and applying pressure to create the tablet, has been reported in the isolation of methadone from water and biosamples [45] and triazine herbicides from water [46]. Recently, Bamorowat et al. proposed a tertiary mixture tablet containing 1-Butyl-3-methylimidazolium hexafluorophosphate [BMIM] [PF_6_], an ionic liquid, as extractant and KBr as binder. After adding the tablet to 5 mL of diluted fruit or vegetable juice, ultrasounds are applied to assist the dispersion of the ionic liquid. After the extraction, the ionic liquid is recovered by centrifugation at the bottom of the extraction tube containing the target analytes (benzoylurea insecticides) [47].

Binary tablets have been proposed for EA-DLLME following two main strategies. On the one hand, the tablet can be prepared only containing the effervescence agents, while the solvent is added in a different step [48,49,50]. On the other hand, the tablet can be prepared containing the carbon dioxide source and the solvent, while the sample is acidified for a better effervescence [51].

#### 3.1.2. Alternative Workflows for EA-DLLME

The use of effervescence in DLLME is very versatile and several workflows, complementary to the previous one, have been proposed.

The own acidity of the sample (e.g., vinegar, fruit juice) can be exploited to simplify the extraction [52,53] since it is not necessary to include the H-donor in the effervescence mixture formulation. The carbon dioxide source can be added to the sample after the solvent addition [52] or the sample can be added to the tube where the carbonate and the solvent have been previously located [53]. Additionally, the H-donor can play a double role. Sorouraddin et al. proposed the use of phthalic acid as both H-donor and chelating ligand for the extraction of Zn and Cd from water samples [54]. Additionally, the use of acidic extractant in solid-liquid extraction is an opportunity for the application of EA-DLLME. Xue et al. proposed the extraction of insecticides from rice using sodium citrate monobasic as one of the extractant elements [55]. After this preliminary extraction, a mixture of undecanol and sodium carbonate aqueous solution is added to isolate the insecticides from the extract.

**Figure 6:**
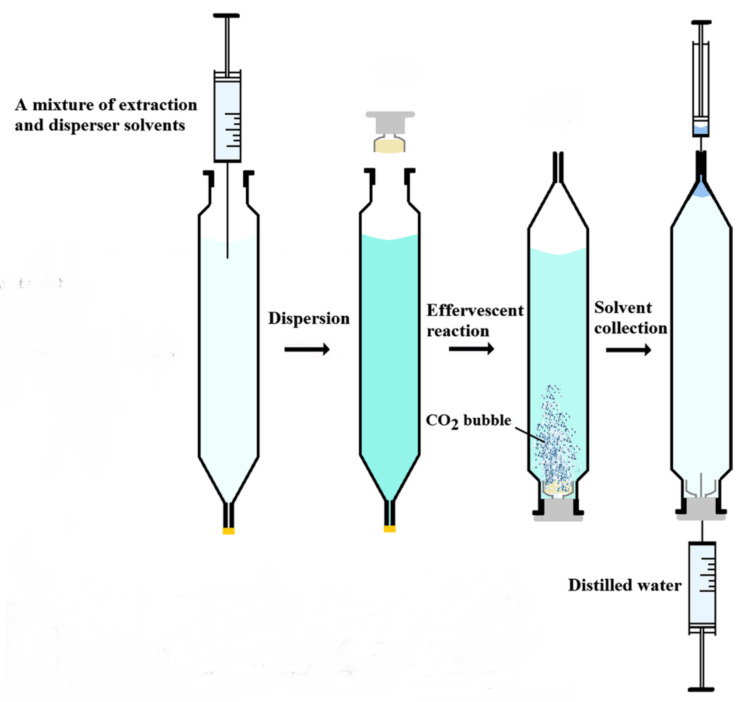
Use of effervescence tablets for phase’s separation in DLLME. Adapted with permission of Springer from reference [56].

Effervescence has also been reported to improve the phase’s separation in DLLME as indicated in Figure 6 [56]. The sample is introduced in a special extraction vessel where the narrower end is closed with a cap. In a subsequent step, the disperser and extractant solvent mixture is injected into the sample leading to a cloudy solution. The wider end of the vessel is then closed with a septum cap containing the effervescent tablet and the vessel is turned while the narrower end is opened. The tablet dissolution creates CO_2_ bubbles that break the dispersion. In the final step, distilled water is injected to move the extractant solvent, lighter than water, to the narrower end where it is collected.

Professor Bulatov’s research group has proposed interesting approaches to the simplification and automation of the technique. In 2016, they described the direct coupling of EA-DLLME with microvolume UV-Vis spectroscopy for the determination of surfactants in water [57]. The method consists of the ion pairing of the surfactants with oppositely charged dyes to form neutral colored species that are finally extracted, aided by effervescence, into the organic solvent (chloroform). The extracts are finally analyzed by UV-vis spectroscopy. The same year, they proposed the first approach for the complete automation of the technique using the determination of antipyrine in saliva samples as the model analytical problem [58]. For this purpose, a stepwise injection analysis system was designed containing a mixing chamber as the core element. Initially, the sample with different derivatization reactions are pumped into the chamber and mixed using a nitrogen stream. After the derivatization, the effervescence precursors (Na_2_CO_3_ and formic acid) and the solvent (dichloromethane) are injected into the chamber to develop the EA-DLLME. This addition is done by a multi-syringe module in such a way that the three chemicals are mixed at in a coil immediately before their application. After the extraction, the organic solvent moves to the lower part of the chamber (specially designed for this purpose) and pumped to the photometer for the detection of the derivatized colored analyte.

### 3.2. Solvents

Apart from the conventional organic solvents, other alternatives have been used in EA-DLLME, including ionic liquids, switchable solvents, and deep eutectic solvents.

Ionic liquids (ILs) are reference solvents in the analytical sample preparation context due to their sorption capacity, low vapor pressure, and tunability (e.g., solubility depending on the anion constituent) [59]. ILs have been used in EA-DLLME [60,61], and their combination with magnetic nanoparticles has permitted the development of the so-called technique magnetic effervescent tablet-assisted ionic liquid dispersive liquid–liquid microextraction (META-IL-DLLME). In a general META-IL-DLLME, the tablet contains the effervescence precursors, the ionic liquid in its hydrophobic form and Fe_3_O_4_ magnetic nanoparticles [62,63]. The liquid sample is placed in a vessel, and the tablet is added. The dissolution of the effervescence precursors induces the formation of CO_2_ bubbles that assist the dispersion of the IL into the sample. The IL is finally retained on the surface of the magnetic nanoparticles that are recovered by an external magnet. In the last step the IL is dissolved in an appropriate solvent for instrumental analysis. Some variations of this general workflow have been reported. Fe_3_S_4_ NPs have been used instead of common Fe_3_O_4_ as their superior surface and porosity provided better sensitivity [64]. In other cases, the IL is added to the tablet in its hydrophilic form, and a metathesis reaction is required to switch the IL to the hydrophobic form that presents an easier recovery. This transformation reaction can be done by adding the metathesis reagent after the tablet dissolution [65] or using it as a tablet ingredient [66]. In other cases, the H-donor is added directly to the sample [67], or the IL plays a double role as extractant and H-donor [68]. Wang et al. have recently proposed magnetic ILs as extractants, which simplifies the overall process as the MNPs are no longer necessary [69].

Switchable solvents (SS) can be defined as those materials that change between two different forms as a response to an external trigger. The use of these solvents in the microextraction context was initially reported by our group in 2015 [70,71] and have been applied to different analytical problems. Although the SS term is usually ascribed to those solvents that change between the hydrophilic and hydrophobic forms depending on the carbon dioxide concentration, other triggers can be applied. In fact, in the EA-DLLME context, fatty acids have been widely reported as SS. Fatty acids switch from the hydrophilic to the hydrophobic states by a simple pH change. In 2017, Shishov et al. proposed the use of SS in EA-DLLME for the extraction of steroid hormones in water samples [72]. In this case, a tablet containing the effervescent precursors (sodium bicarbonate and oxalic acid) and the solvent (sodium nonate) is prepared. After the addition of two tablets to the water sample, the effervescence reaction takes place, giving rise to two consecutive reactions. In the first one, sodium bicarbonate reacts with oxalic acid driving to sodium oxalate, water and carbon dioxide. In the second reaction, the sodium nonate reacts with an excess of oxalic acid, driving to sodium oxalate and nonaoic acid. Nonaoic acid is finally recovered as a second phase containing the target analytes. Some slight variations of this general workflow have been reported. Gao et al. [73] and Hemmati and Rajabi [74] have proposed an alternative workflow based on the separate additions of the effervescence precursors as liquid reagents, doing the final separation of the fatty acid by the solidified drop technique [73] or simple decantation [74]. A similar approach, but using tables has been proposed for the determination of endocrine-disrupting compounds in bottled water [75]. As previously indicated in META-IL-DLLME the solvent recovery can be improved if magnetic nanoparticles are added to the tablet [76].

Deep eutectic solvents (DES) are considered as a class of ionic liquids as they share with them some properties like low volatility, high extraction capacity, high viscosity, and tunable properties depending on their chemical composition. DES are synthesized by the combination of an H-bond acceptor (HBA) and an H-bond donor (HBD), which results in a substance with a lower melting point (eutectic mixture) than the individual precursors. DES have clear potential in Analytical Chemistry [77], and their solvent related properties can be exploited for sample treatment [78,79]. In 2018, two different groups proposed the use of DES in EA-DLLME [80,81]. Ravandi and Fat’hi applied a workflow very similar to the first EA-DLLME application as the DES dissolved in acetic acid is directly added to the sample, which is previously spiked with sodium bicarbonate [80]. In contrast, Arpa et al. used an effervescent tablet to assist the mixing between the DES and the sample [81]. As it occurs with other solvents, magnetic nanoparticles [82] and the solidification technique [83] have been proposed for the separation of the DES after the extraction. Very recently, Shishov et al. have proposed an interesting approach based on the use of DES in EA-DLLME [84]. In this case, the HBD of the DES acts as the proton donor in the effervescent reaction, and the HBA acts as the solvent.

## 4. Other Uses of Effervescence-Assisted Extraction

Effervescence-assisted microextraction is a very versatile technique as it can be adapted to different formats (including solid-phase and liquid-phase approaches) and sample types (including liquid and solid ones). The main contributions to the technique have been presented and discussed in this article and summarized in Table 1. However, the versatility of effervescence has also been exploited in other sample treatment approaches.

**Figure 7:**
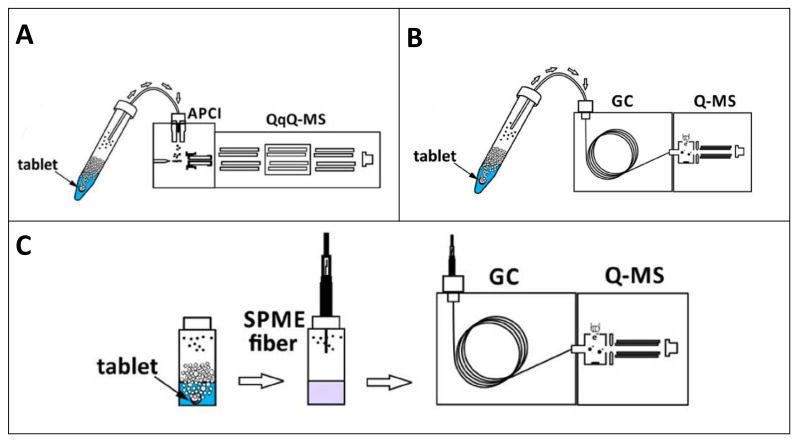
Use of effervescence tablets for fizzy extraction. (**A**) Coupling with mass spectrometry; (**B**) coupling with gas chromatography; and (**C**) coupling with solid-phase microextraction. Adapted with permission of the American Chemical Society from [88].

Fizzy extraction consists of the purge of volatile compounds from a sample using a CO_2_ stream [85]. The technique can be coupled directly to a detector [86], but it is also compatible with a previous chromatographic separation [87]. Urban et al. have recently applied the effervescence reaction to fizzy extraction [88]. The tablet’s dissolution generates CO_2_ bubbles that drag the volatile and semi-volatile compounds from the sample matrix. The flexibility of the approach is easily observable in Figure 7. Figure 7A shows how the analytes can be transferred to a mass spectrometer using an Atmospheric-pressure chemical ionization (APCI) as ionization source. Figure 7B presents the direct combination with Gas Chromatography-Mass Spectrometry while Figure 7C describes the coupling of fizzy extraction with SPME. In the latter case, the CO_2_ bubbles transfer the analytes from the sample to the headspace, from where they are finally extracted into the fiber.

Garcia-Barrera et al. have recently reported the combination of effervescence with headspace hollow fiber LPME [89]. A unique design is proposed to make this arrangement compatible. The hollow fiber, containing the organic solvent as the acceptor, is spin around to a dedicated cap to avoid its direct contact with the sample.

## Figures and Tables

**Table 1:** Selected applications based on the use of effervescence-assisted microextraction.

Effervescent Agents		Extractant	Sample		Analytes	Notes	Ref
CO_2_ Donor	H Donor		Type	Amount			
Na_2_CO_3_	NaH_2_PO_4_	Oasis HLB	Water	10 mL	Nitroaromatic compounds	The tablet is placed on the syringe used as extraction vessel. Effervescence occurs upon sample aspiration inside the vessel. Sorbent with extracted analytes is recovered by syringe filter.	[9]
Na_2_CO_3_	NaH_2_PO_4_	MWCNTs	Water	100 mL	Triazines	Nanotubes are only effectively dispersed in tablet format with no additional organic solvent.	[16]
Na_2_CO_3_	NaH_2_PO_4_	G-MWCNTs-COOH	Hawthorn herb	200 mL	Natural antioxidants	The tertiary tablet is prepared by blending the ingredients and applying pressure. Different types of nanotubes were evaluated.	[18]
Na_2_CO_3_	NaH_2_PO_4_	Mesoporous hybrid materials (PCMA-60)	Root extracts	20 mL	Tanshinones	The tertiary tablet is prepared by blending the ingredients and applying pressure. Higher amounts of sorbent (13 mg) produce aggregation and decrease in extraction efficiency	[19]
Na_2_CO_3_	NaH_2_PO_4_	β-cyclodextrin/attapulgite composite	Water	7 mL	Pyrethroids	The tablet is placed on the syringe used as extraction vessel. Effervescence occurs upon sample aspiration inside the vessel. Sorbent with extracted analytes is recovered by syringe filter	[20]
Na_2_CO_3_	NaH_2_PO_4_	Magnetic attapulgite/polypyrrole nanocomposites	Honey	100 mL (diluted)	Pyrethroids	The tertiary tablet is prepared by blending the ingredients and applying pressure. Magnetic properties of the sorbent are used to facilitate sorbent recovery.	[21]
Na_2_CO_3_	NaH_2_PO_4_	IL-Magnetic-β-cyclodextrin/attapulgite composite	Honey and Juice	8 mL	Fungicides	The tertiary tablet is prepared by blending the ingredients and applying pressure. The sorbent is easily recovered using an external magnet.	[22]
Na_2_CO_3_	NaH_2_PO_4_	NiFe_2_O_4_ MNPs	Seafood extracts	30 mL	Heavy metals	The tertiary tablet is prepared by blending the ingredients and applying pressure. The sorbent is easily recovered using an external magnet.	[24]
Na_2_CO_3_	Citric acid	Fe_3_O_4_/chitosan-Se MNPs	Sausage extracts and Water	10 mL	Heavy metals	The tertiary tablet is prepared by blending the ingredients and applying pressure. The sorbent is easily recovered using an external magnet. Selenium increase extraction potential for metal ions	[25]
Na_2_CO_3_	Citric acid	Dopamine-modified magnetic graphene oxide	Sausage extracts and Water	100 mL	Metal ions	The tertiary tablet is prepared by blending the ingredients and applying pressure. The sorbent is easily recovered using an external magnet. Dopamine enhances extraction capacity.	[26]
Na_2_CO_3_	Citric acid	Dopamine-carbon graphite nitride nanosheets	Oil and water samples	100 mL	Metal ions	The tertiary tablet is prepared by blending the ingredients and applying pressure. Sorbent with extracted ions is separated by centrifugation.	[27]
Na_2_CO_3_	Citric acid	Fe_3_O_4_@SiO_2_@N_3_ MNPs	Urine and pharmaceutical wastewater	10 mL	Antidepressant drugs	The tertiary tablet is prepared by blending the ingredients and applying pressure. The sorbent is easily recovered using an external magnet. Nitrogen-rich surface increases adsorption capacity.	[29]
Na_2_CO_3_	Tartaric acid	Ni-based N-doped Graphene tubes	Deproteinized milk	5 mL	Bisphenols	The tertiary tablet is prepared by blending the ingredients and applying pressure. Magnetic properties of Ni-based tubes are used to facilitate sorbent recovery.	[30]
Na_2_CO_3_	NaH_2_PO_4_	Core-shell magnetic COF	Water, beverages and biosamples	5 mL	Endocrine disruptors	The tertiary tablet is prepared by blending the ingredients and applying pressure. Magnetic properties of the sorbent are used to facilitate sorbent recovery.	[31]
NaHCO_3_	NaH_2_PO_4_	benzo-15-crown-5	*C. fraxini* medicinal plant	-	Coumarins	The procedure consists of matrix solid-phase dispersion extraction.	[32]
NaHCO_3_	NaH_2_PO_4_	CNT /polystyrene-divinylbenzene composite	Biosamples	1 mL (for liquid samples) and 6 mL (for reconstituted solid samples)	Alkaloids and flavonoids	The extractant is prepared as effervescent powder (sodium bicarbonate) inside a pipette tip. The proton donor is added to the aqueous sample before manual withdrawal. The effervescence occurs inside the pipette tip dispersing the sorbent.	[33]
NaHCO_3_	NaH_2_PO_4_	IL-coated core-shell SiO_2_@Fe_3_O_4_ MNPs	Plasma	10 mL (diluted)	Betablockers	Synthesized sorbent (IL-SiO_2_@Fe_3_O_4_) added separated to effervescent precursors shows better extraction efficiency than adding the components mixed (non-immobilized IL).	[34]
NaHCO_3_	NaH_2_PO_4_	[3C_6_C_14_P] [BF_4_]	Water	10 mL	Benzoylurea insecticides	A tertiary tablet containing the effervescence precursors and the IL is prepared. After the extraction, the solvent is recovered as a solid in the upper part of the centrifugation tube.	[60]
Na_2_CO_3_	NaH_2_PO_4_	Ionic liquid nanofluid	Honey and tea	8 mL (honey is 1:10 *w*/*v* diluted)	Acaricide	A tertiary tablet containing the effervescence precursors and the IL nanofluid is prepared. The solvent is recovered by centrifugation.	[61]
Na_2_CO_3_	NaH_2_PO_4_	[HMIM] [PF_6_]	Food samples	10 mL (pretreated sample)	Selenium	The ionic liquid is added to the tablet that also contains the effervescence precursors and magnetic nanoparticles.	[62]
Na_2_CO_3_	NaH_2_PO_4_	[HMIM] [NTf_2_]	Water	8 mL	Fungicides	The ionic liquid is added to the tablet that also contains the effervescence precursors and magnetic nanoparticles.	[63]
Na_2_CO_3_	NaH_2_PO_4_	[BMIM] [PF_6_]	Water and milk	7 mL (pretreated sample)	Polybrominated diphenyl ethers	The ionic liquid is added to the tablet that also contains the effervescence precursors and magnetic nanoparticles Fe_3_S_4_ are used instead of common Fe_3_O_4_.	[64]
Na_2_CO_3_	Tartaric acid	[HMIM] [BF_4_]	Urine and serum	7 mL (diluted and pretreated sample)	Endogenous steroids	The ionic liquid is added to the tablet that also contains the effervescence precursors and magnetic nanoparticles After the extraction NH_4_PF_6_, is added to make the IL recovery easier.	[65]
Na_2_CO_3_	NaH_2_PO_4_	[BMIM]_2_ [Br]_2_	Meat	5 mL (pretreated sample)	Polycyclic Aromatic Hydrocarbons	The tablet contains the effervescence precursors, the IL, the metathesis reagent, and the magnetic nanoparticles NiFe_2_O_4_ nanoparticles are used.	[66]
Na_2_CO_3_	HCl	[HMIM] [PF_6_]	Milk	8 mL (pretreated sample)	Pyrethroids	The ionic liquid is added to the tablet that also contains the CO_2_ source and magnetic nanoparticles. HCl is added previously to the sample. The magnetic nanoparticles simplify the IL recovery as it coats the surface of the nanomaterial.	[67]
NaHCO_3_	[BMiM][HSO_4_]		Tea beverage	5 mL	Triazine herbicides	The IL acts as solvent and H+ donor. After the extraction, NH_4_PF_6_ is added to make the IL recovery easier. The IL is recovered by centrifugation.	[68]
Na_2_CO_3_	NaH_2_PO_4_	[BMIM][FeCl_4_],	Vegetables	10 mL (pretreated sample)	Arsenite and arsenate	The tablet contains the effervescence precursor and the magnetic IL.	[69]
NaHCO_3_	Oxalic acid	Sodium nonate	Water	1 L	Steroids	The tablet contains the effervescence precursor and the solvent. Two tablets are added to the sample	[72]
Na_2_CO_3_	Sulfuric acid	Fatty acid	Several samples	10 mL (pretreated sample)	Antibiotics	The effervescence precursors are added as solutions The fatty acid is recovered by the solidification of floating drop technique.	[73]
Na_2_CO_3_	Sulfuric acid	Fatty acid	Food samples	6 mL (pretreated sample)	Azo dyes	The effervescence precursors are added as solutions.	[74]
NaHCO_3_	Citric acid	Sodium octanoate	Beverage	5 mL	Endocrine disrupting chemicals	The tablet contains the effervescence precursor and the solvent. The fatty acid is recovered by the solidification of floating drop technique.	[75]
NaHCO_3_	Citric acid	Sodium hexanoate	Water	5 mL	Triazine herbicides	Magnetic nanoparticles are added to the tablet to aid the recovery of the sample after the extraction.	[76]
NaHCO_3_	Acetic acid	DES containing Aliquot 336 and decanoic acid	Food	8 mL (pretreated sample)	Synthetic dyes	DES is dissolved in acetic acid and injected into the sample containing NaHCO_3_.	[80]
Na_2_CO_3_	NaH_2_PO_4_	DES containing choline chloride and phenol	Water	25 mL	Copper	An effervescent tablet is place in the extraction vessel. Later, the sample and the DES are introduced into the vessel.	[81]
NaHCO_3_	Citric acid	DES containing hexyltrimethylammonium bromide and 1-dodecanol	Water	5 mL	EDC	Fe_3_O_4_ coated with activated carbon nanoparticles is added to recover the solvent after the extraction.	[82]
NaHCO_3_	Citric acid	DES containing thymol with octanoic acid	Liquid samples	5 mL	Fungicides	The DES is recovered by solidification of DES.	[83]
Na_2_CO_3_	Formic acid	DES containing formic acid and menthol	Liver	10 mM (pretreated sample)	Ketoprofen, diclofenac	The HBD acts as proton donor in the effervescent reaction while the HBA acts as the solvent.	[84]
